# Supplementary material for: A bimodal soft electronic skin for tactile and touchless interaction in real time
Source: Nat Commun. 2019 Sep 27;10:4405. doi: 10.1038/s41467-019-12303-5 (PMC6764954; doi:10.1038/s41467-019-12303-5)
Supplement: Supplementary file 1 — Supplementary Information [file 41467_2019_12303_MOESM1_ESM.pdf]

Supplementary Information

A bimodal soft electronic skin for tactile and touchless interaction in real time

Ge et al.

Supplementary figures

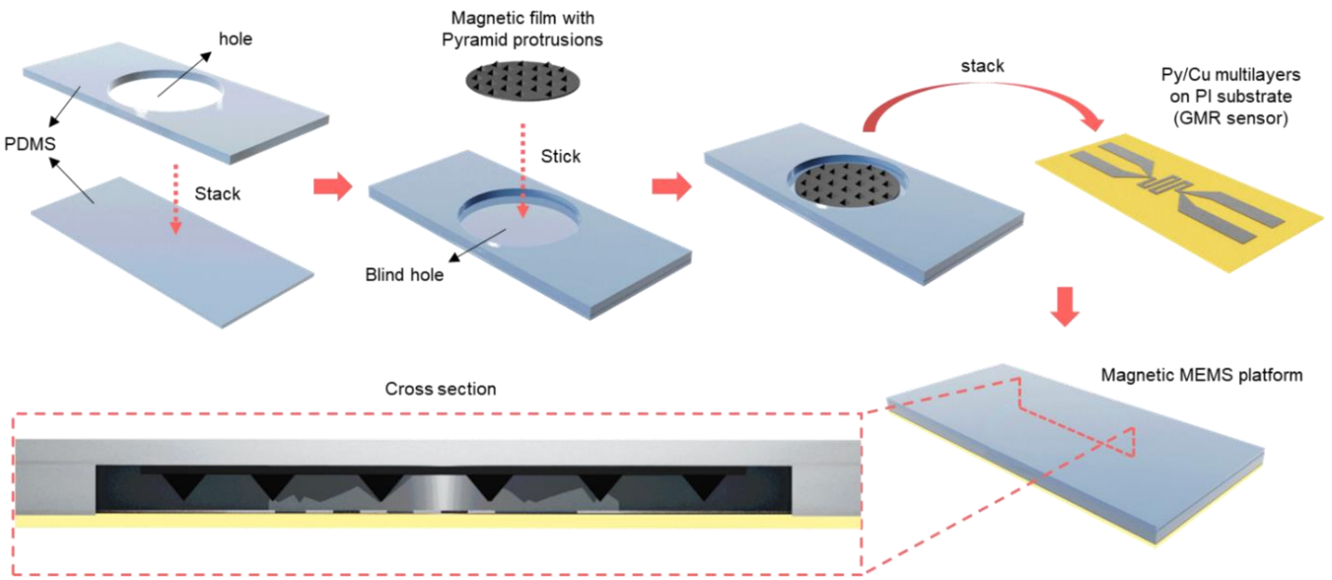

Supplementary Figure 1. Fabrication flow of the m-MEMS platform. PDMS films of different thickness were fabricated using a film applicator (TOC AB3400). A PDMS frame with a blind hole was fabricated by laminating a PDMS film (210 µm thick) with a hole in the center to a homogeneous PDMS film with a thickness of 115 µm. The hole with a diameter of 5 mm was made by a hole puncher. In the next step, a compliant permanent magnet (NdFeB microparticles embedded in PDMS) with pyramid-shaped extrusions was magnetized in an in-plane magnetic field of 1.5 T. A piece with a dimeter of 4 mm was cut out of this composite film using a hole puncher. This circular patch was placed in the blind hole of the PDMS frame. Afterward, the PDMS frame was laminated to a GMR sensor (Py/Cu multilayers) prepared on a PI foil. The meander pattern of the GMR sensor was positioned right below the compliant permanent magnet.

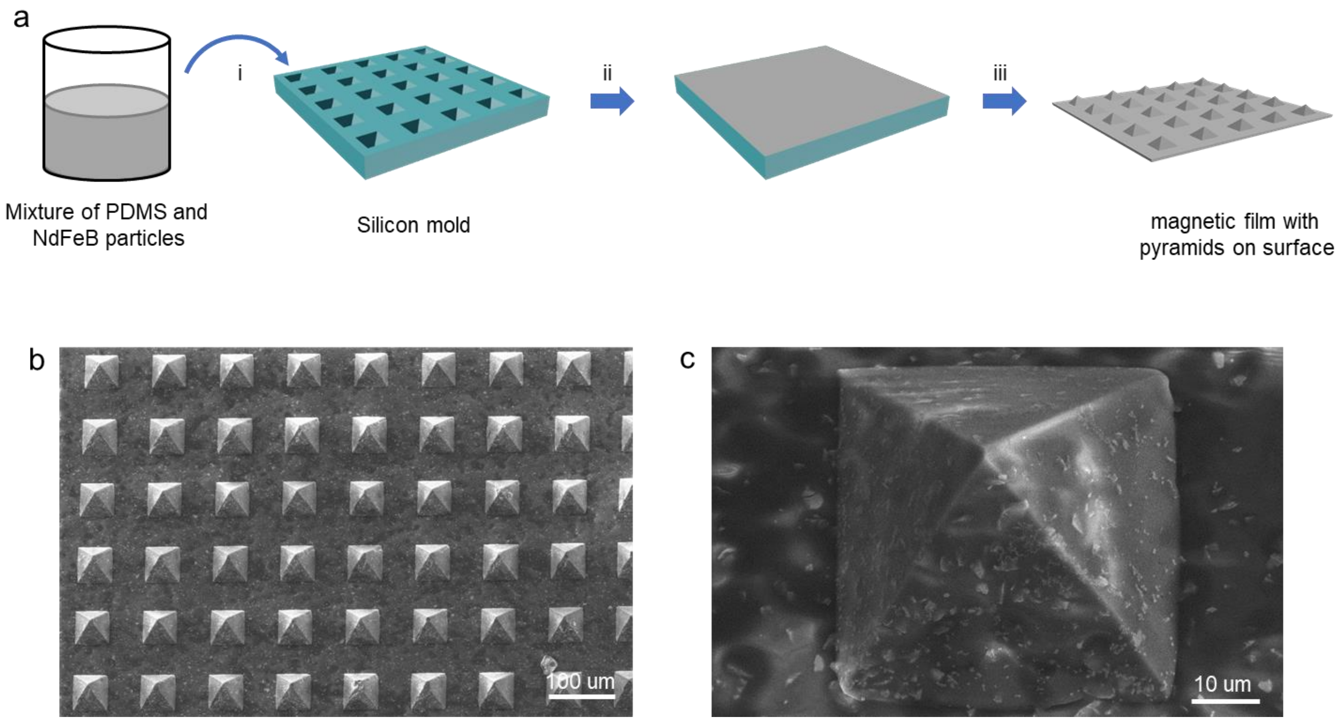

Supplementary Figure 2. a Fabrication process of a compliant permanent magnet with pyramid-shaped extrusions. (i) PDMS (Sylgard 184, precursor to curing agent is 10:1) and NdFeB microparticles are mixed in a weight ratio of 3:7. Then, the mixture is drop casted onto a silicon mold and degassed under vacuum. (ii) The silicon mold was heated at 80°C on a hotplate for 1 h to cure the NdFeB/PDMS composite. (iii) The compliant permanent magnet was gently peeled off from the silicon mold. **b** and **c** SEM images of the surface of the compliant permanent magnet. The base plane of the pyramid is 50 x 50 μm^2. The height of the pyramid is about 35 μm. The distance between adjacent pyramids is about 100 μm. The number of the pyramids on the built-in magnet is about 1255.

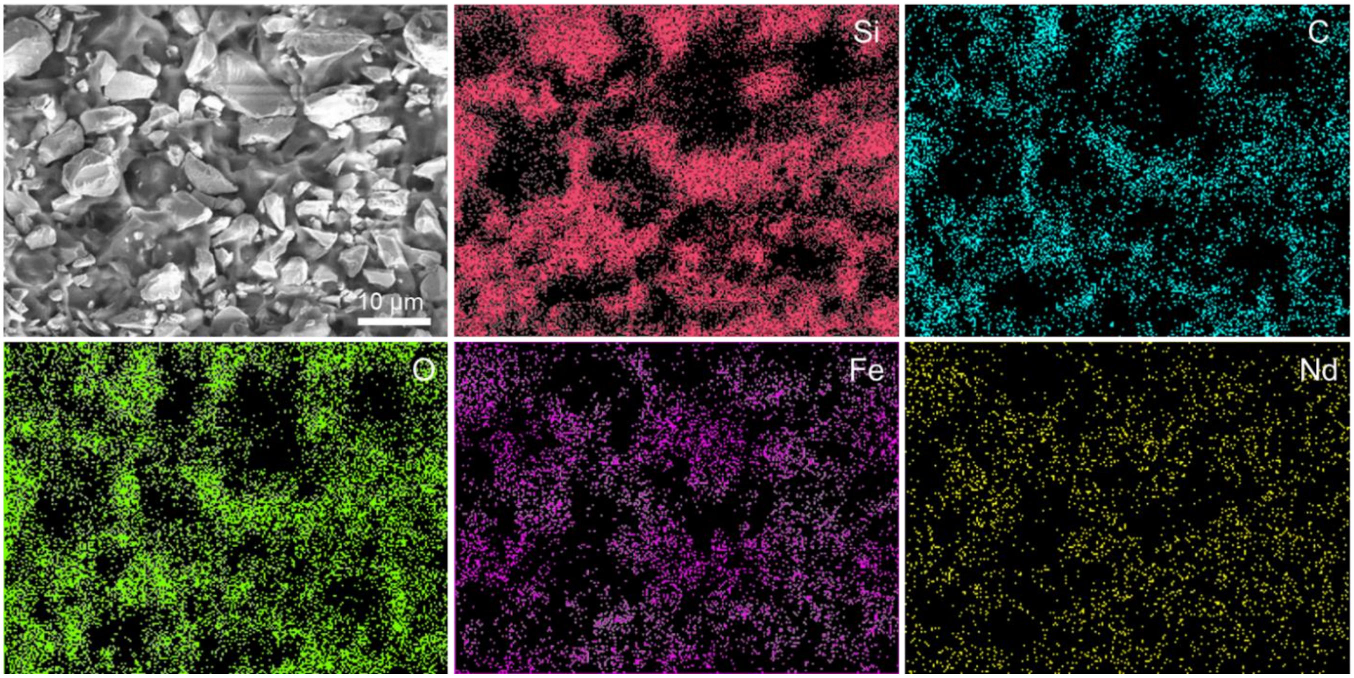

Supplementary Figure 3. Elemental mapping (EDX) of the cross-section of the compliant permanent magnet. The elements Si, C, and O are from the PDMS rubber. The elements Fe and Nd are from NdFeB microparticles. These elemental mapping images show that NdFeB microparticles are homogeneously embedded in the PDMS matrix.

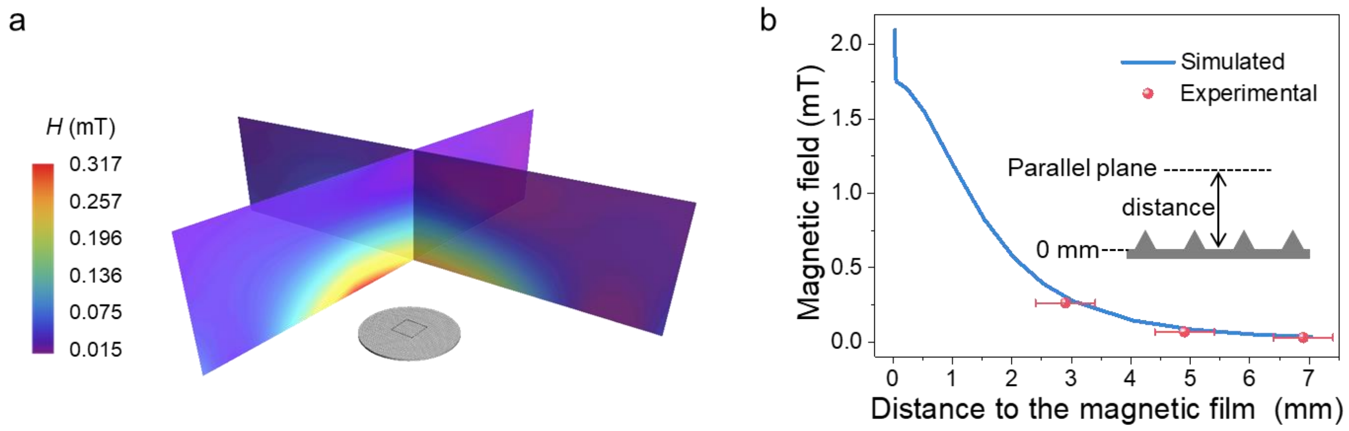

Supplementary Figure 4. a A magnetic stray field profile at the top of a compliant permanent magnet measured by a Gauss meter (HGM09s). **b** The simulated dependence of the averaged magnetic stray field as a function of the distance from the top surface of the compliant permanent magnet. The experimental data points are shown with red symbols.

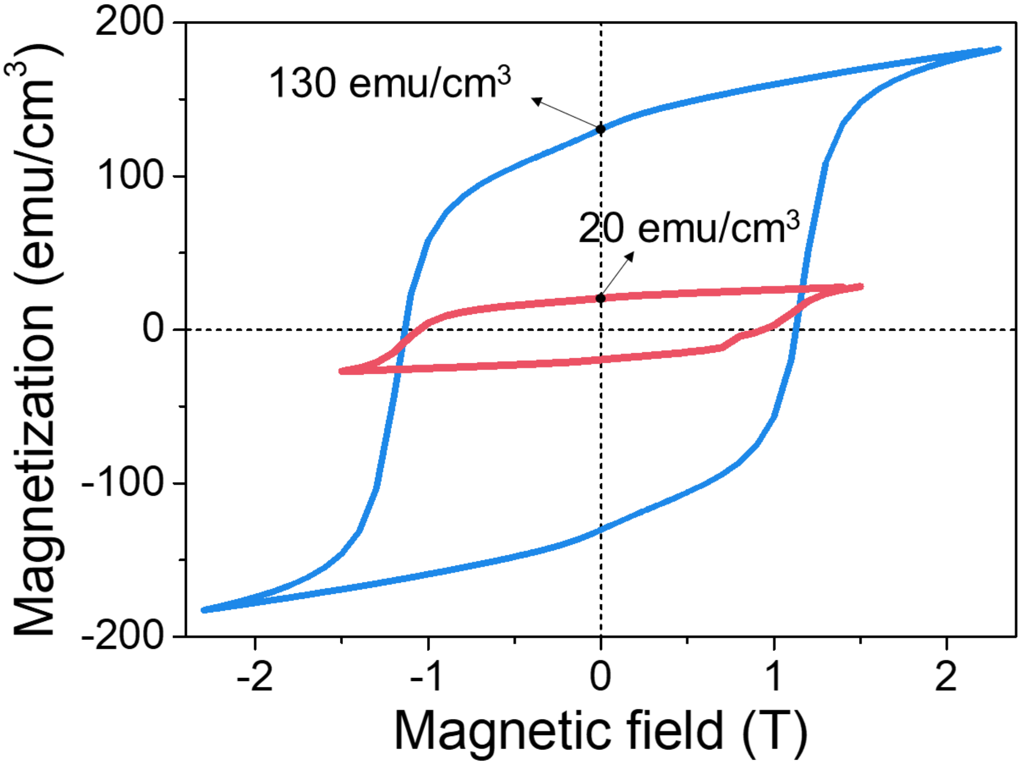

Supplementary Figure 5. Magnetic hysteresis loops of a compliant permanent magnet with pyramid-shaped extrusions measured using SQUID-VSM at room temperature between ± 1.5 T (red curve) and ± 2.5 T (blue curve). The remanent magnetization is 20.5 emu cm^{-3} (when the field is cycled between ± 1.5 T; red curve) and 130.5 emu cm^{-3} (when the field is cycled between ± 2.5 T; blue curve). Prior to the measurement, the compliant permanent magnet with pyramid-shaped extrusions was magnetized in an in-plane magnetic field of 1.5 T.

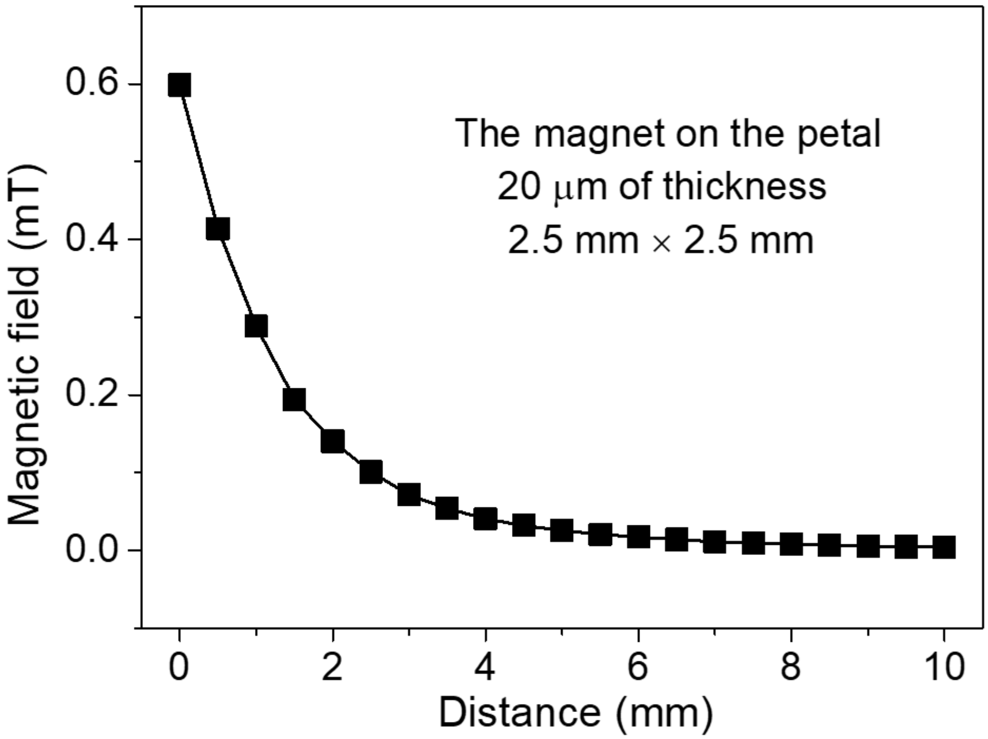

Supplementary Figure 6. The change of the normal component of a magnetic stray field of a compliant permanent magnet (20 µm thick; lateral dimensions: 2.5 × 2.5 mm^2), located on a flower petal, as a function of distance. The distance is measured from the center of the square shaped magnet along its surface normal. The magnet has the same composition as the compliant magnet of the MEMS platform, but without pyramid-shaped extrusions. Prior to the measurements, the magnets used for these studies were magnetized in an in-plane magnetic field of 2.3 T. They possess a remanent magnetization of about 130 emu cm^{-3} (Supplementary Figure 5).

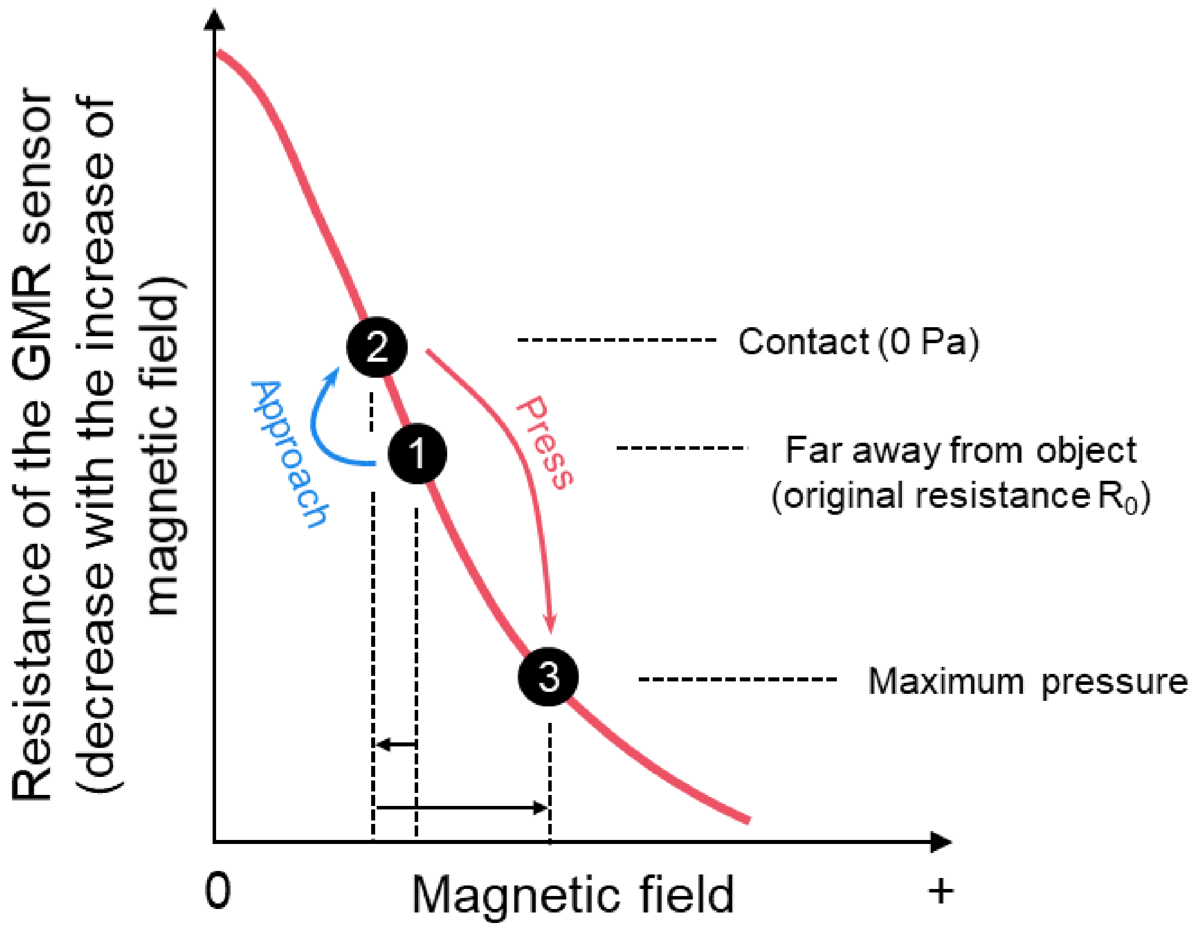

Supplementary Figure 7. The change of the electrical resistance of a GMR sensor in an external magnetic field is shown with the red line. When embedded into the m-MEMS platform, the GMR sensor is biased by the magnetic field of the built-in compliant permanent magnet. This defines the initial value of electrical resistance of the GMR sensor on the red curve. Typically, the region of highest sensitivity is chosen, which is indicated with the point ①. Upon touchless interaction with a magnetic object (possessing the magnetic field orientation, which is opposite to the orientation of the built-in magnetic field of the MEMS platform), the magnetic field at the sensor location will decrease and the resistance will become smaller, reaching the point indicated as ② in the ultimate case when the sensing platform touches the object. In this process, the change of the electrical resistance (current value minus the value without external magnetic field (= point ①)) will be negative. The lowest negative value of the resistance change is reached when the touch event happens. With further tactile interaction, the built-in magnet will approach to the sensor, which will result in the increase of the magnetic field at the sensor location. Remarkably, the magnetic field will surpass the initial biasing magnetic field (for details, see Supplementary Figure 8). This will cause the electrical resistance to become larger than its initial value. Therefore, the change of the electrical resistance (current value minus the value without external magnetic field (= point ①) will become positive. The largest resistance (indicated as point ③) and the largest change of the resistance is reached upon the strongest press. The qualitative change of the signal (negative upon touchless interaction and positive upon tactile interaction) allows unambiguous discrimination between two interaction modes.

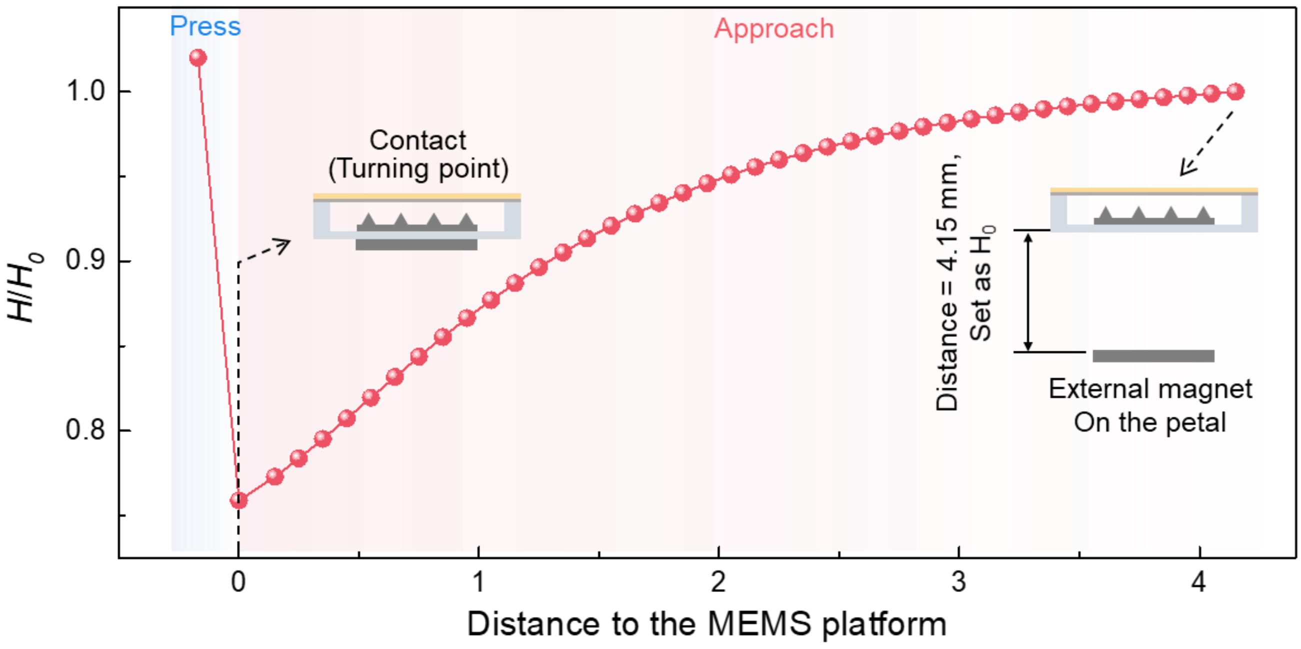

Supplementary Figure 8. Relative change of the magnetic field (H/H_0) at the location of the magnetic field sensor when the m-MEMS platform approaches and presses an external magnet located on a flower petal (Supplementary Figure 6). H_0 denotes the magnetic field strength at the distance between the sensor and a magnet of 4.15 mm.

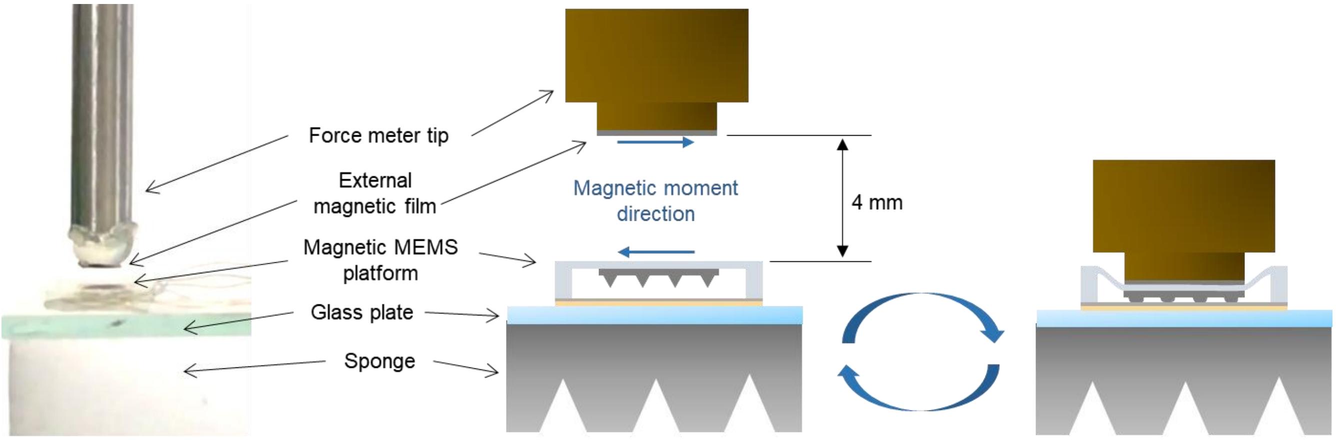

Supplementary Figure 9. The measurement configuration for distinguishing tactile and touchless sensing modes. A piece of a compliant permanent magnet (the same magnetization as that of the magnetic on the petal, Supplementary Figure 6; 4 mm in diameter, 20 µm thick) is immobilized at the tip of a force meter. The direction of the magnetic field provided by this external source is chosen to be opposite to that of the built-in compliant permanent magnet. Initially, the distance between the external magnet and the top surface of the m-MEMS platform is 4 mm. The m-MEMS platform moves forward to the external magnet until both objects are in a physical contact. Then, a pressure between the external magnet and the m-MEMS platform is applied. When the pressure reaches a certain value, the pressure is released, and the m-MEMS platform moves back to its initial position. The resistance of the m-MEMS platform and the pressure applied to it are recorded during the entire process. The pressure is measured using a force gauge (Sauter, FH 5N). The pressure is applied to the m-MEMS platform by moving it up to press the tip of the force gauge. The motion of the m-MEMS platform is controlled using a motorized stage (neMESYS 290N). The m-MEMS platform is fixed to a glass substrate. To apply pressure slowly, a commercial elastic sponge was used as a buffer layer to support the glass substrate.

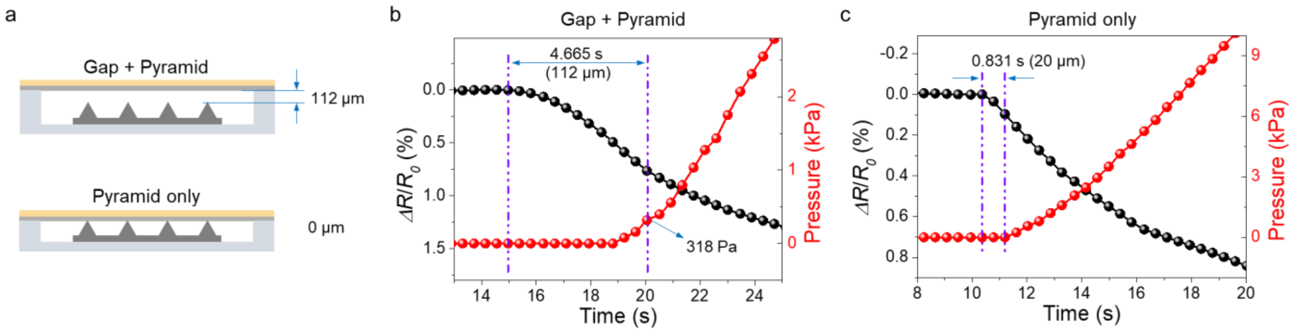

Supplementary Figure 10. a Schematic illustration of air gap of two different device configurations. The upper panel in (a) shows the case with the distance between a pyramid tip and a GMR sensor of 112 µm. **b** The resistance change (black symbols) of the device containing both air gap and pyramids, and the pressure change (red symbols) of the force meter during the process of pressing. **c** The resistance change (black symbols) of the device containing pyramids only and the pressure change (red symbols) of the force meter during the process of pressing. In (a) and (c), the devices were moved up at a speed of 24 µm s^{-1}.

The gap height between a pyramids tip and GMR sensor is designed to be about 110 µm. In the measurement of the pressure sensing performance, the force meter is fixed. To determine the pressure sensing performance, the force meter is fixed and only the devices are able to move up towards the force meter at a speed of 24 µm/s. The force meter has a resolution of 1 mN corresponding to 79 Pa. At the beginning of the pressing process, the pressure is too small to be detected by the force meter. However, the resistance change of the sensor tells us when the pressure sensor started to be pressed. Supplementary Figure 10b shows the resistance change of the device (with air gap and pyramids) and the pressure change at the force meter. From the curves, we see that the pressure value did not change when the resistance of the GMR sensor began to decrease. This indicates that the pressure at the initial stage of the pressing process is very small (at least smaller than the pressure detection limit of 79 Pa of the force meter). But from the turning point of the resistance change (indicated by the left dash-dotted line), we could determine that this point corresponds to the time, when the device got in contact with the force meter. The second turning point (indicated by the right dash-dotted line) of the resistance change indicates that the pyramids began to contact the GMR sensor. The time between two turning points is 4.665 s, which corresponds to 112 µm of displacement.

For the device with pyramids only, the force meter again is unable to detect the small pressure at the initial stage of the pressing process (Supplementary Figure 10c). After about 20 µm displacement, the pressure sensor indicated an increase of pressure. Therefore, for the device with air gap and pyramids, the estimated distance is 112 µm – 20 µm = 98 µm, which is close to the designed gap of 112 µm. In this case, the mutual contact between the pyramid tips and GMR sensor happened before the second turning-point of the pressure change in Supplementary Figure 10b. In other words, the pressure at the moment of contact between a pyramid tip and GMR sensor is smaller than ~ 318 Pa. This value is slightly higher than the simulated result shown in Fig. 3b (~764 Pa).

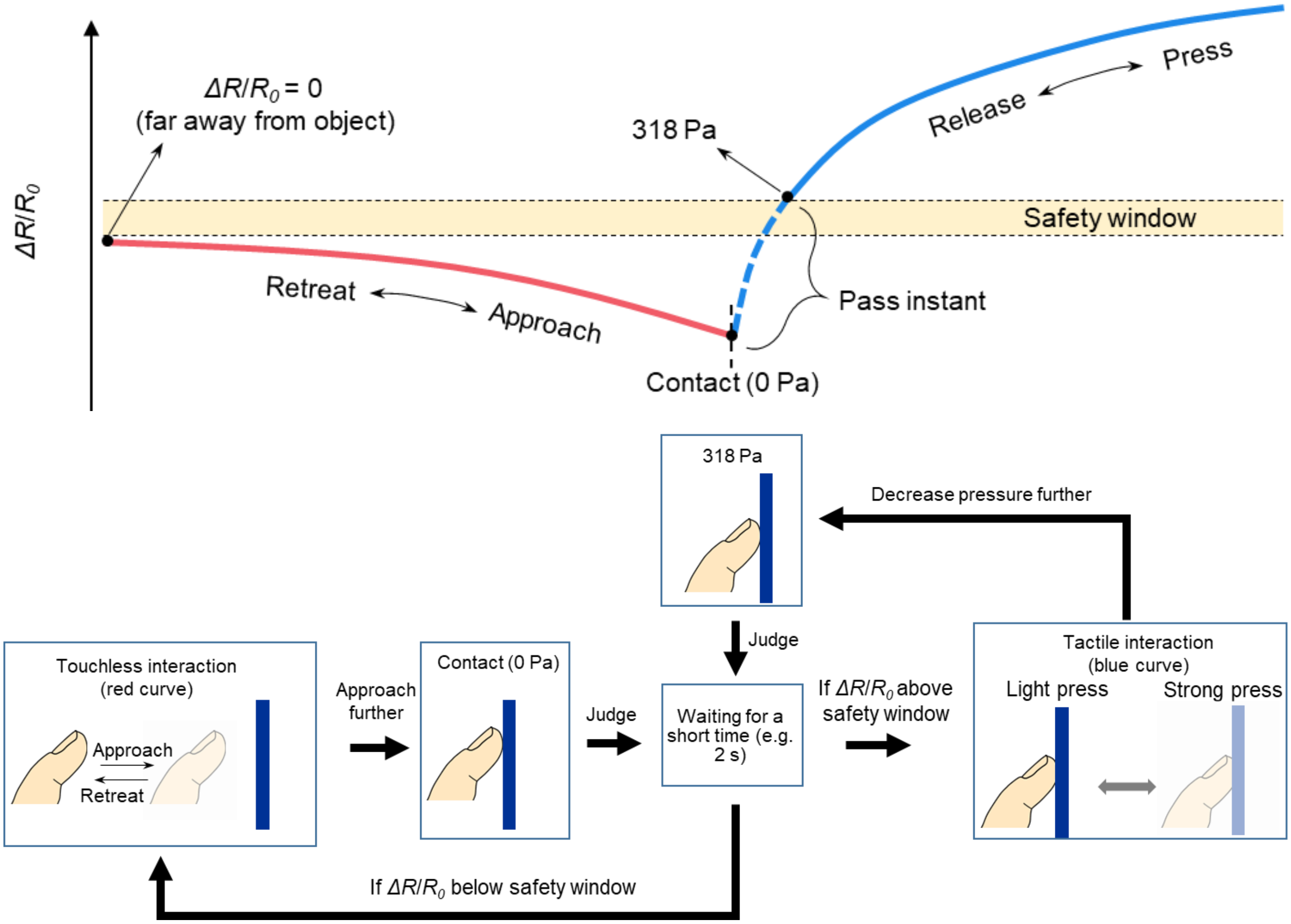

Supplementary Figure 11. Algorithm for the discrimination of the two interaction modes (sensor is mounted on the fingertip). Upon approaching or retracting the finger bearing an m-MEMS to or from an external magnetic object (blue object), the electrical resistance of the magnetic field sensor integrated in the m-MEMS platform will change (see Supplementary Figure 7). As soon as the change of the electrical resistance, $\Delta R/R_0$, reaches its lowest value, the event is treated as a physical touch of finger and object. Up to this point, the signal follows the red curve in the upper panel. The softest touch recognized by humans is about 1 kPa. Therefore, a press of a user will quickly bring the signal of the sensor to the value above the safety window and the event will be recognized as a pressing event. To avoid any possible misjudgment, after reaching the contact point, the logic of the device makes a pause for a certain user-defined time. During this waiting time no decision is made by the device based on the sensor signal. This gives the user a chance to either retract the finger (after the waiting time, this action will be interpreted as a retreat motion while touchless interaction) or proceed with pressing (after the waiting time, this action will be interpreted as tactile interaction). The decision regarding the actual interaction mode is made based on the level of the signal measured after the waiting time: if the signal is above the safety window, the interaction is assigned to be tactile (the signal changes along the blue curve in the upper panel). If after the waiting time, the signal level is below the safety window, the device will interpret the interaction as touchless (the signal changes along the red curve in the upper panel).

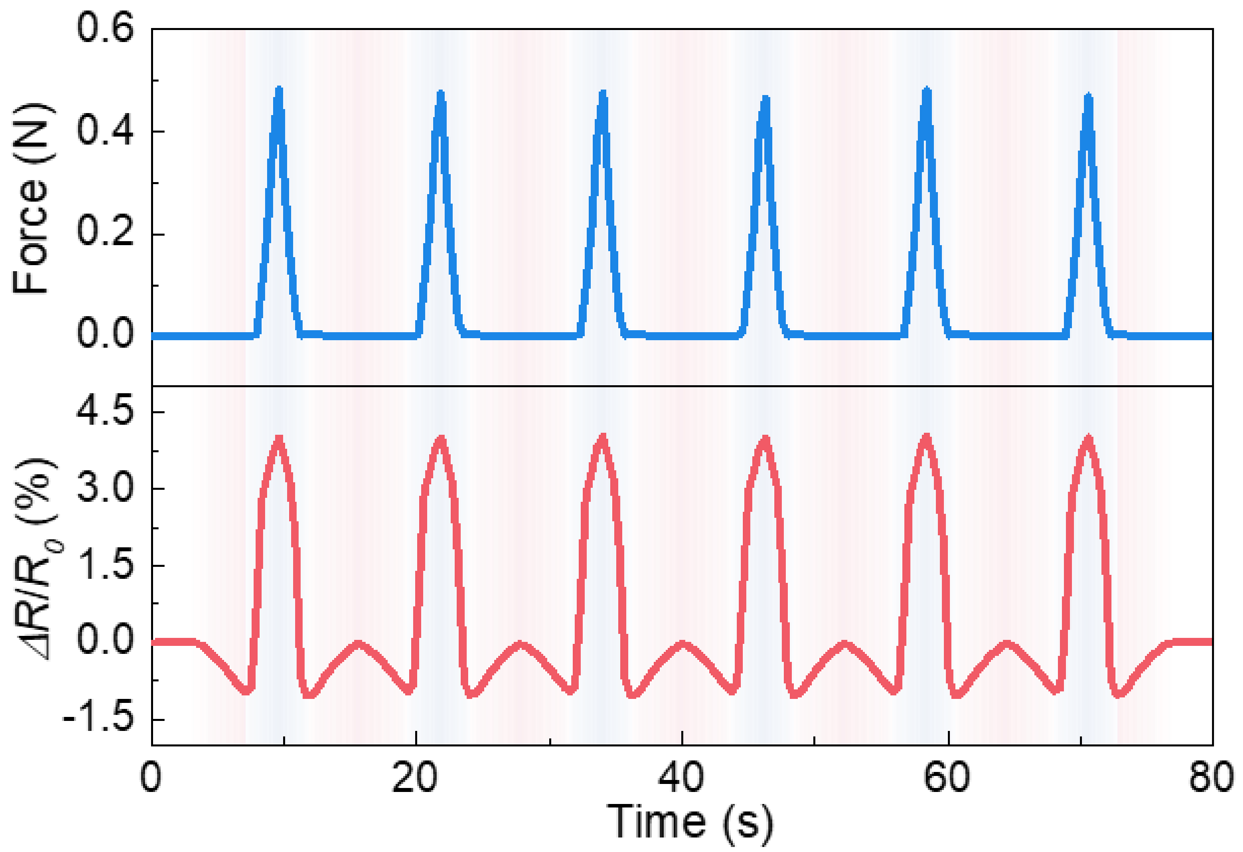

Supplementary Figure 12. The interaction events as in Fig. 2g repeated six times.

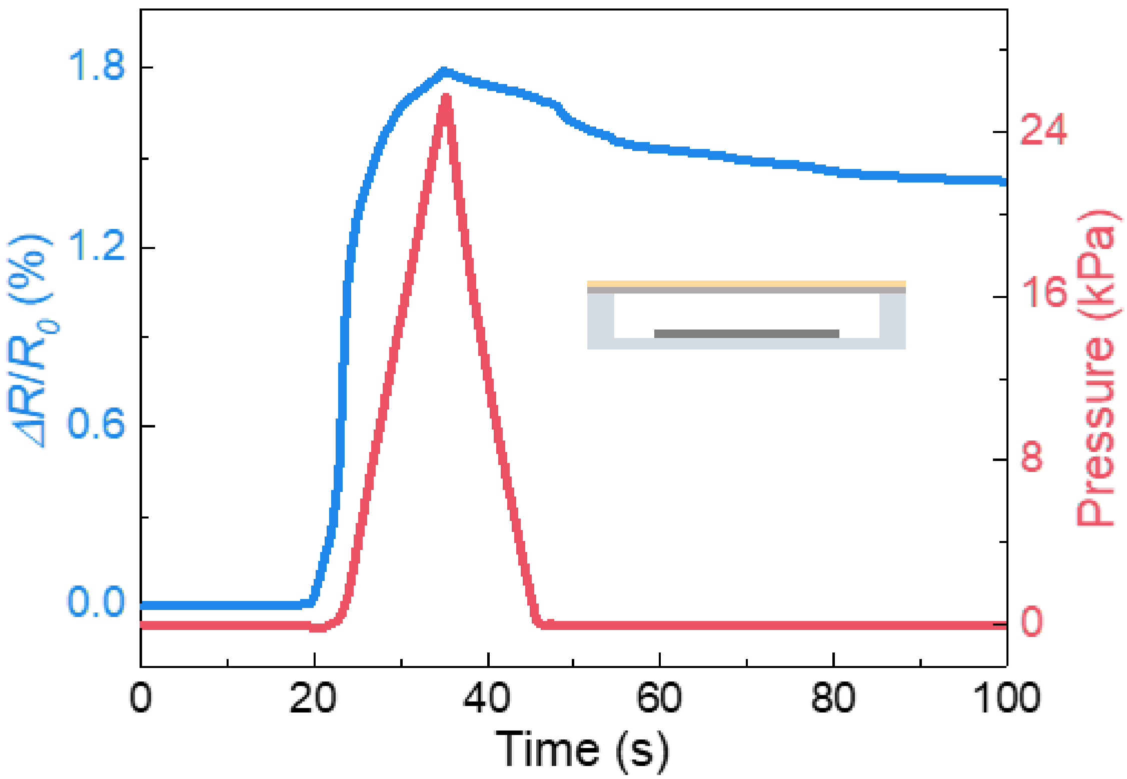

Supplementary Figure 13. The experimentally determined change of the electrical resistance of the sensor with an air gap only (no pyramids) in the press-release test.

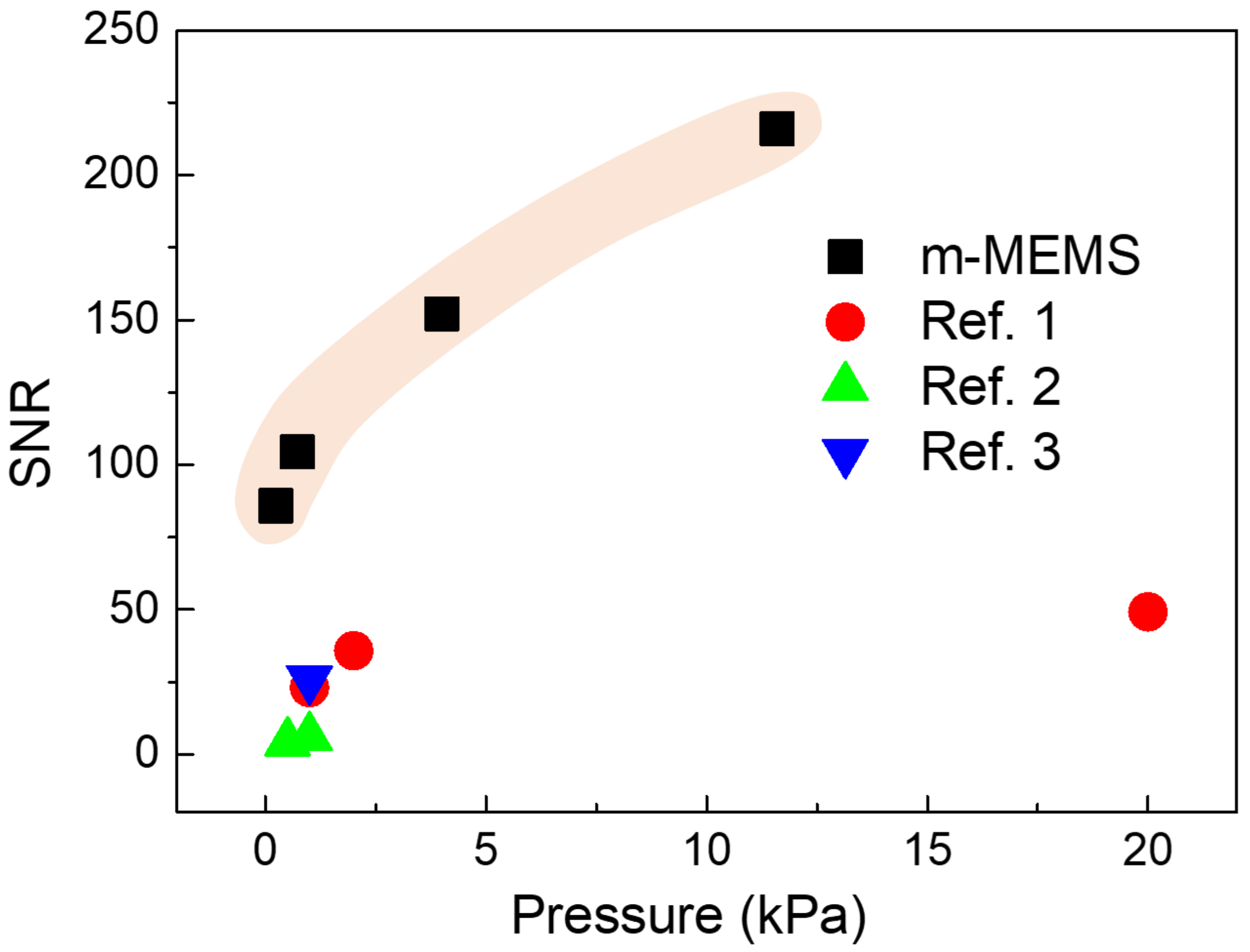

Supplementary Figure 14. The signal-to-noise ratio (SNR) of the compliant m-MEMS platform versus pressure. A comparison to the state of the art (Supplementary References (*1*-*3*)) is given as well. The value of noise (N) is extracted by measuring fluctuations of the baseline (the difference between the maximum value and minimum value in the baseline). The signal of the sensors (S) in response to a certain pressure is the difference between the mean value of the response signal and the mean value of the baseline. The SNR is defined as a ratio between signal (S) and noise (N). The SNR of Supplementary References (*1*-*3*) are estimated based on the data provided in Figs. 2b, 2c and 3a of (Supplementary Reference *1*), data in Fig. 2a, Supplementary Figure 3 of (Supplementary Reference *2*), data in Figs. 2b and 2d of (Supplementary Reference *3*). The SNR of the m-MEMS is substantially higher than the state of the art reports. This is the key parameter, which determines the sensor performance. We note that amplifying the signal is technically easier the higher the SNR.

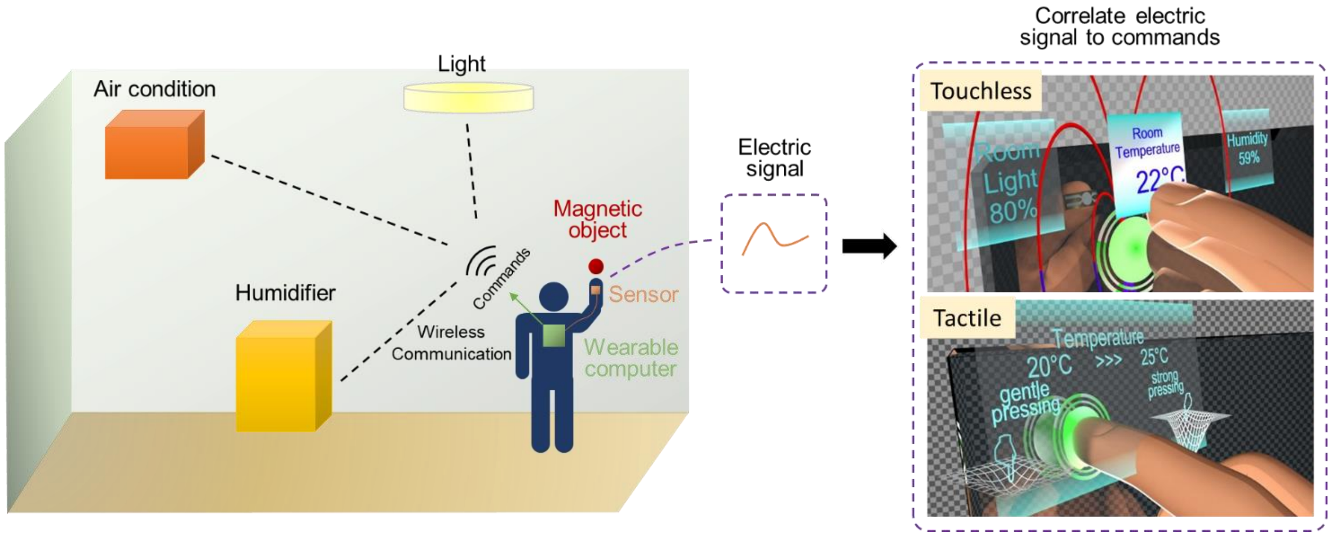

Supplementary Figure 15. The application concept of the m-MEMS platform in augmented reality settings. The magnetic object of interest works like a virtual knob, which enables the interaction with a physical object, e.g. light, humidifier or similar device. The magnetic knob is located conveniently for its manipulation. When our m-MEMS platform approaches and presses the magnetic object, the electric signals will be analyzed by a wearable computer and be transformed to commands (i.e. select the device of interest, change the value). The commands will be sent wirelessly to the device of choice (e.g. air conditioner) to change the actual setting of the device, say the temperature in the room.

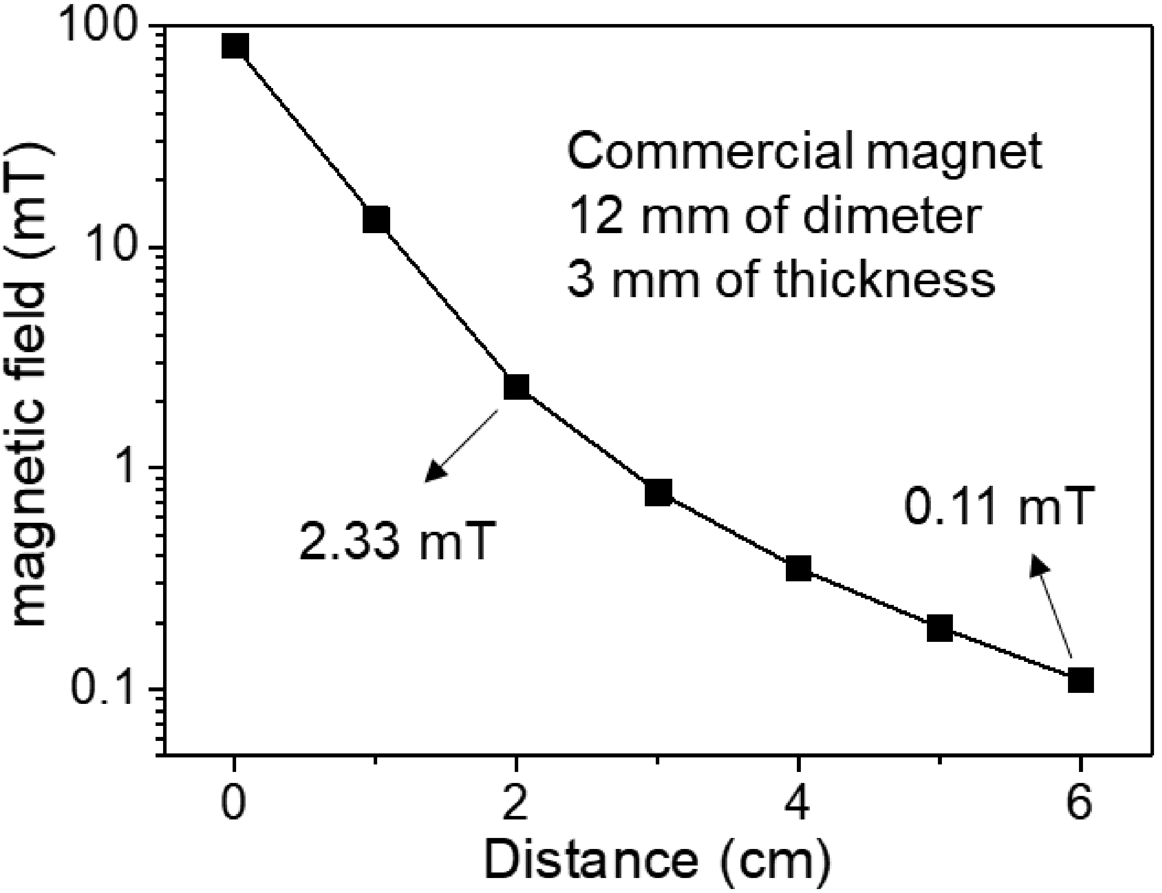

Supplementary Figure 16. The change of the normal component of a magnetic stray field of a commercial magnet (used for measurements shown in Fig. 5; 3 mm thick; diameter: 12 mm) as a function of the distance. The distance is measured away from the surface of the magnet along the line parallel to the symmetry axis of the cylindrical magnet.

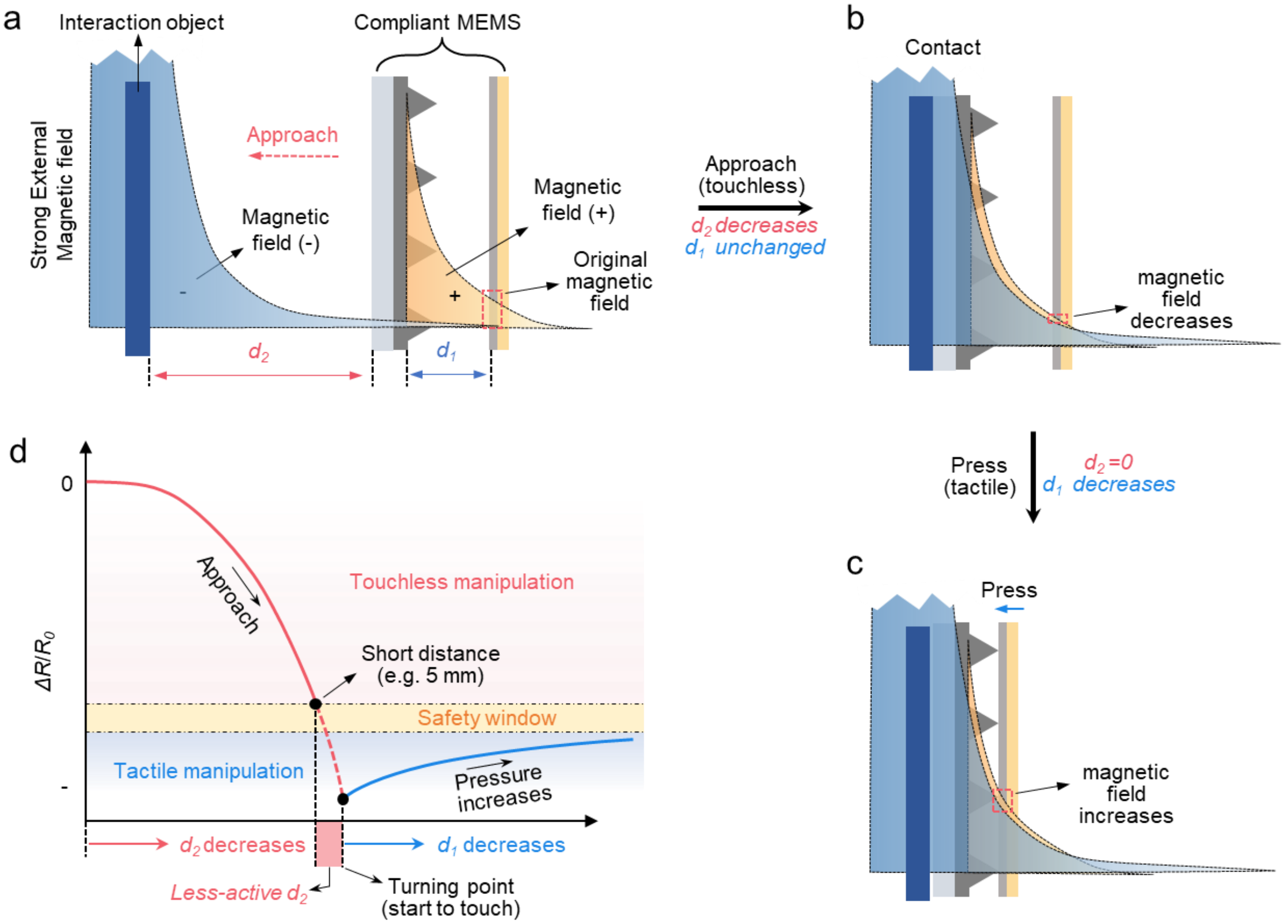

Supplementary Figure 17. a Schematic illustration of the compensation of the built-in magnetic field, provided by a compliant permanent magnet (orange-shaded region), using an external magnetic field source (blue-shaded region). The resulting magnetic field at the location of the magnetic field sensor (Py/Cu multilayers) is relevant for the discussion. The compliant m-MEMS platform interacts with a magnetic object, which exposes the m-MEMS to an external magnetic field. Firstly, the strength of the external magnetic field at the sensor location decreases with the decrease of the distance between the external magnet and the m-MEMS platform (indicated as distance d_2 in panel (a)). When the magnetic object approaches the m-MEMS platform, its built-in magnetic field can be compensated (either partly or completely) by the external magnetic field. Therefore, the magnetic field sensor will be exposed to a different magnetic field upon approaching the external magnet, which will lead to the modification of its electrical resistance. This aspect is crucial to enable the touchless interaction (Fig. 2h). Considering the peculiarity of the GMR effect, the change of the electrical resistance ($\Delta R/R_0$) will increase (becomes more negative) upon approaching an external magnetic object given that its stray field is opposite to the built-in magnetic field of the m-MEMS platform. This is indicated with a solid red line in **d**. The signal will continue decreasing up to the point when the object will be brought in contact with the m-MEMS platform as illustrated in **b**. This will correspond to the smallest signal level shown in **c**. Further approach of the object to the sensor will necessarily lead to the application of pressure to the m-MEMS platform (panel **c**) and the sensor will operate in the tactile mode. The evolution of the $\Delta R/R_0$ is illustrated in **d**.

In the case of a strong external magnetic field, the total variation of $\Delta R/R_0$ during the touchless interaction (red curve) is larger than that during the tactile interaction (blue curve). Therefore, the signal acquired upon the tactile interaction only slightly changes the signal acquired upon the touchless interaction. In this case, the algorithm of the discrimination of the interaction mode is as follows: The signal strength ($\Delta R/R_0$) above the safety window (solid red curve) can be correlated to a group of commands for the manipulation of objects in a touchless mode. The signal strength below the safety window (the blue curve) can be correlated to a group of commands for the manipulation of objects in a tactile mode. The upper limit for the safety window is defined as the signal that is achieved when the finger is placed at a certain distance above the object. From the convenience point of view, it is not easy for humans to manipulate objects in a touchless mode when the distance between the MEMS platform and the object is small, e.g. 5 mm in the case of our demonstrator. This bound is pre-set accordingly to the preference of the user. The lower limit for the safety window is determined as the signal corresponding to the event of the strongest touch. This bound is defined by the design of the m-MEMS platform. The signal between these two bounds (within the safety window) is excluded from the analysis. The signal above the safety window is considered as a touchless interaction. The signal below the safety window is considered as a tactile interaction.

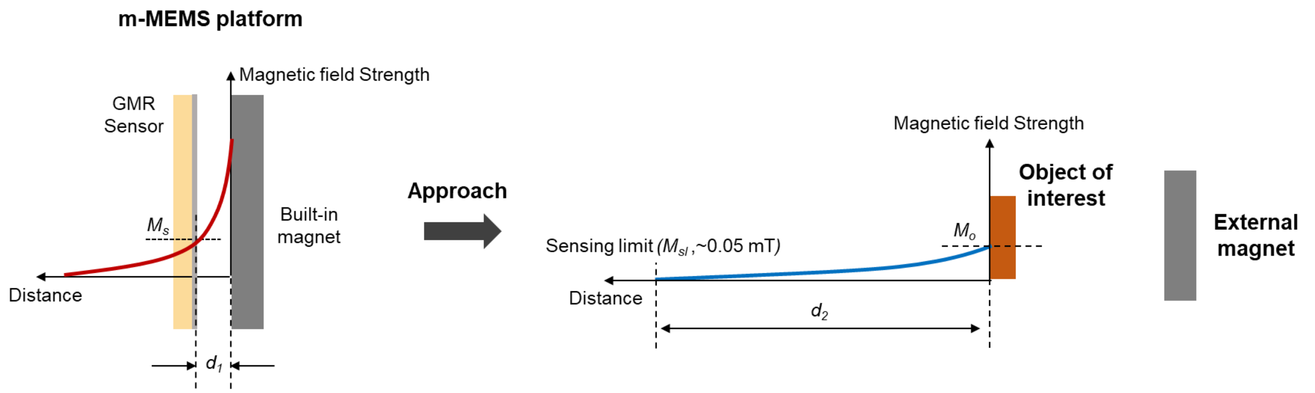

Supplementary Figure 18. Schematic illustration of the key factor that determines the value of d_2. The value of d_2 can be tuned in a wide range depending on the use case. For example, d_2 is about 5 mm for the flower petal in Fig. 2 of the main text, and is about 30 mm in Fig. 5. The value of d_2 is determined by the gradient of the magnetic field of the object. As shown in this figure, the magnetic field at the plane of the object's surface (H_0) should be smaller than the magnetic field at the plane of the GMR sensor (H_s). d_2 is the distance between the surface of the object and the plane where the magnetic field is lowered to the sensing limit of the GMR sensor (named as H_{sl}, which is in our specific case of about 0.05 mT). Therefore, d_2 increases with the decrease of the gradient between H_0 and H_{sl}. The gradient of the magnetic field can be tuned by choosing a proper design of a magnet or using arrays of magnets to shape the stray field at will.

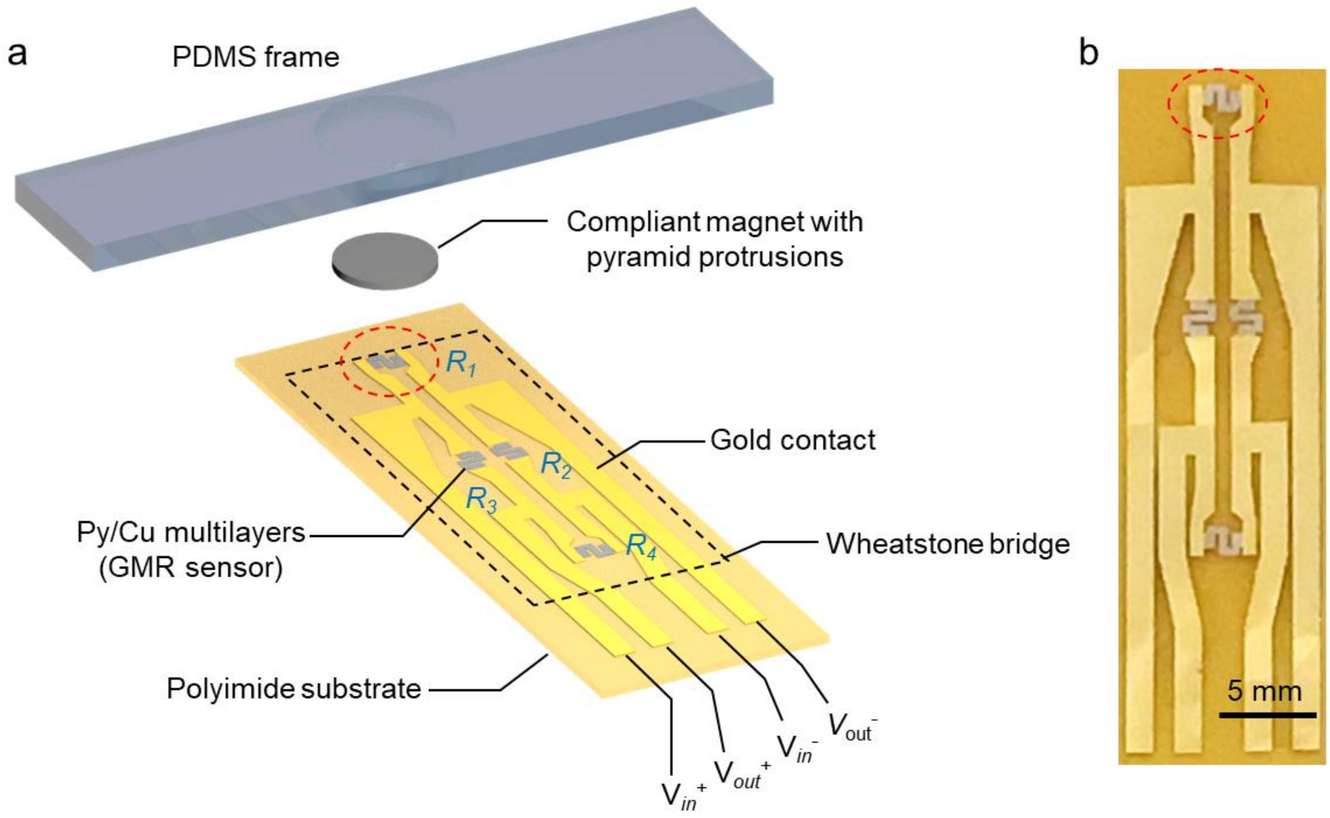

Supplementary Figure 19. a Schematic illustration of the compliant m-MEMS platform using a Wheatstone bridge (composed of four GMR sensors) instead of a single GMR sensor. R_1, R_2, R_3, R_4 are the resistances of the GMR sensors indicated in the panel (a). These resistances are nominally the same when no magnetic field is applied to the device. The GMR sensor indicated with a red circle is the only one integrated into an m-MEMS platform. It is biased by a compliant permanent magnet with pyramid-shaped extrusions. This GMR sensor is located closest to the fingertip. Hence, it will be also the one affected most by the proximity of an external permanent magnet. In this case, the change of the resistance of this GMR sensor will be bigger than the other three GMR sensors, leading to an unbalancing of the Wheatstone bridge [$R_1 < R_2 = R_3 = R_4$]. The arrangement with a Wheatstone bridge not only improves the sensitivity of the magnetic field sensor but also suppresses signal drifts caused by temperature variations. **b** Photograph of a compliant Wheatstone bridge consisting of 4 GMR sensors.

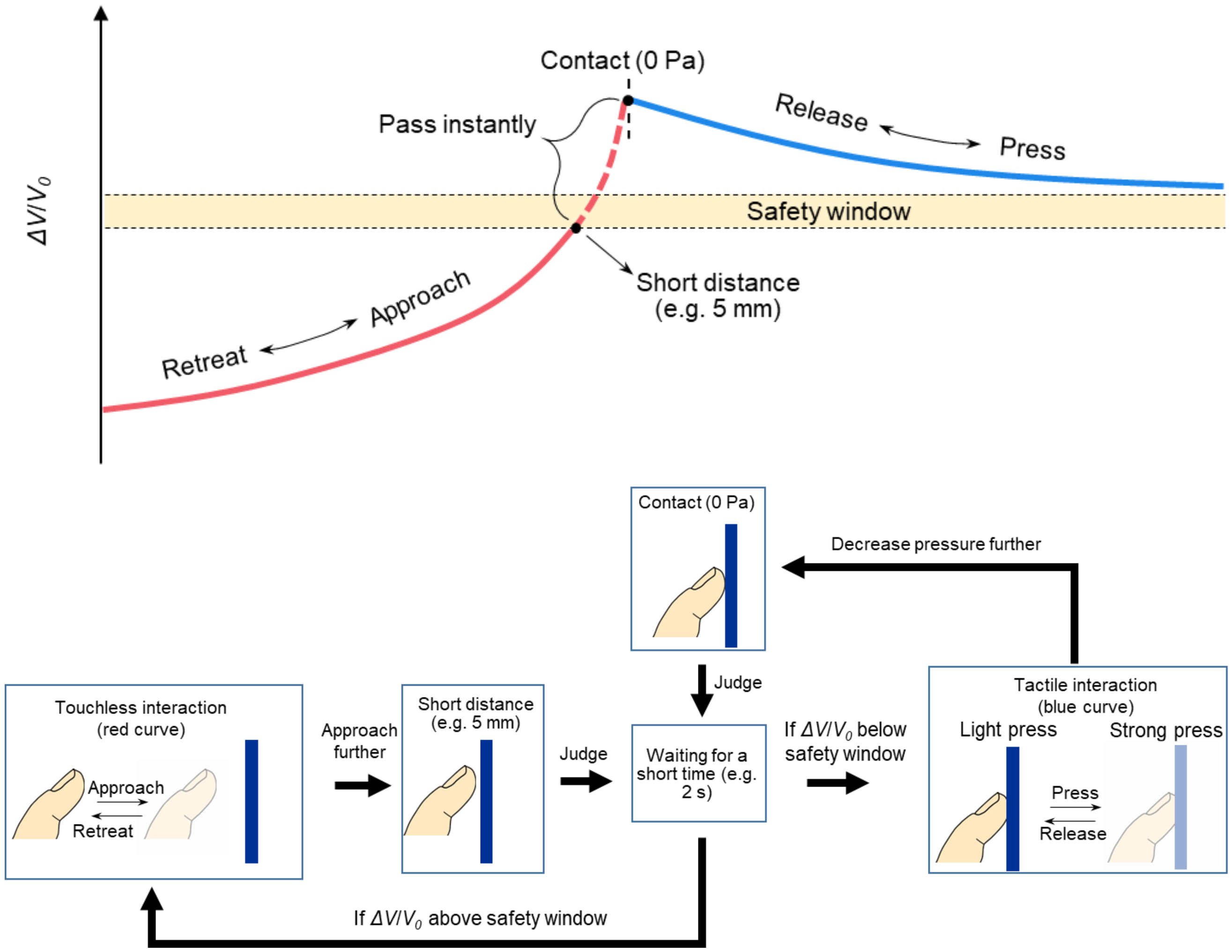

Supplementary Figure 20. Algorithm of the discrimination of the two interaction modes in the case when the m-MEMS platform is included into a Wheatstone bridge. The change of the signal is shown in the upper panel of the figure. The background of the signal change is discussed in the caption of Supplementary Figure 17. Following the same logic as for the case of the single sensor (see discussion of the Supplementary Figure 11), the separation between touchless and tactile modes is based on the signal level with respect to the defined safety window (orange-shaded region in the upper panel of this figure). The touchless interaction is performed up to the distance smaller than a certain "short distance" (custom-defined in our demonstrator to be 5 mm). The signal corresponding to the manipulation of virtual objects follows the red line. When the touchless interaction is finished and the user wants to switch to the tactile interaction, the finger will approach quickly toward the magnetic object and press it. As soon as the signal reaches the value at the defined short distance of 5 mm, the acquired signal will not be analyzed for a certain defined waiting time. After this waiting time, the signal will be analyzed: if the signal level will be below the safety window, the interaction mode will be assigned to be touchless. If the signal level is above the safety window, the operation mode will be considered as tactile.

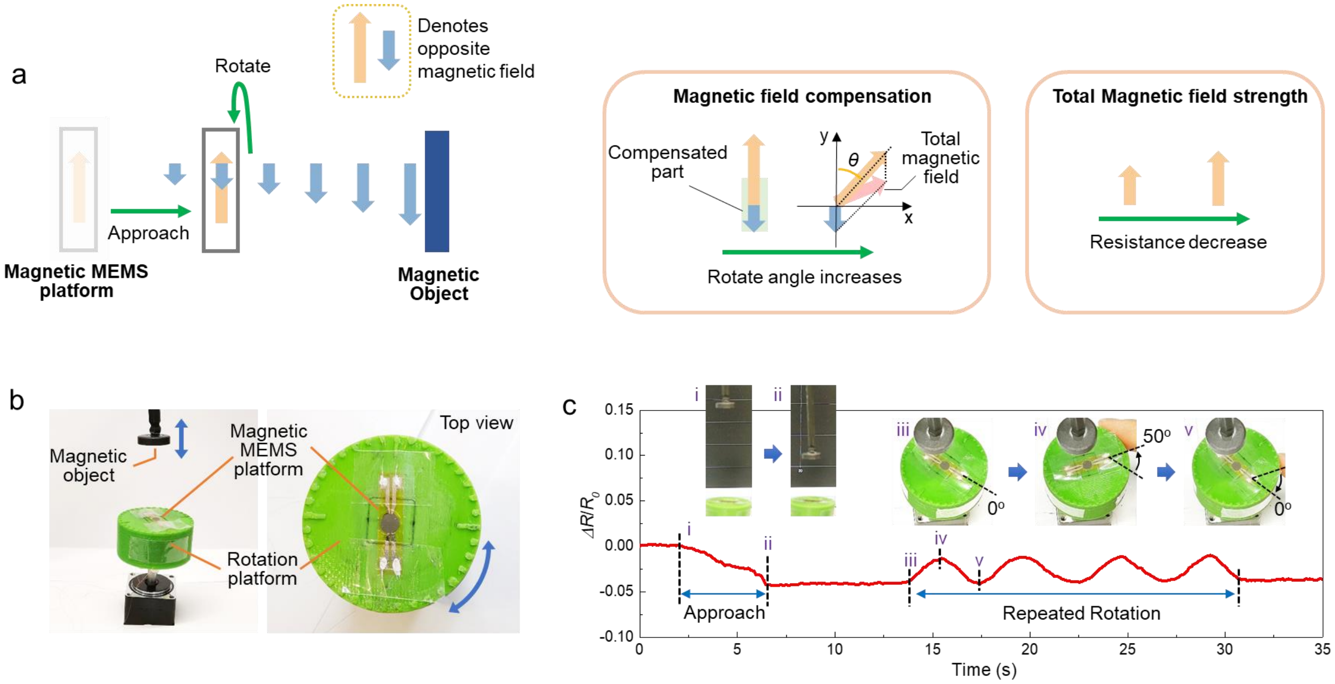

Supplementary Figure 21. a Schematic illustration of the compensation effect of the built-in magnetic field upon rotating the m-MEMS platform with respect to the stray field of a magnetized object. **b** A setup to study the angle sensing functionality of the m-MEMS platform. **c** The change of the electrical resistance of the compliant GMR sensor upon approaching to and rotating with respect to a permanent magnet. R_0 denotes the initial resistance of the GMR sensors. $\Delta R = R_0 - R$, where R is the actual value of the electrical resistance when the sensor is exposed to an external magnetic field. The information shown in this figure is correlated with the Supplementary Movie 5.

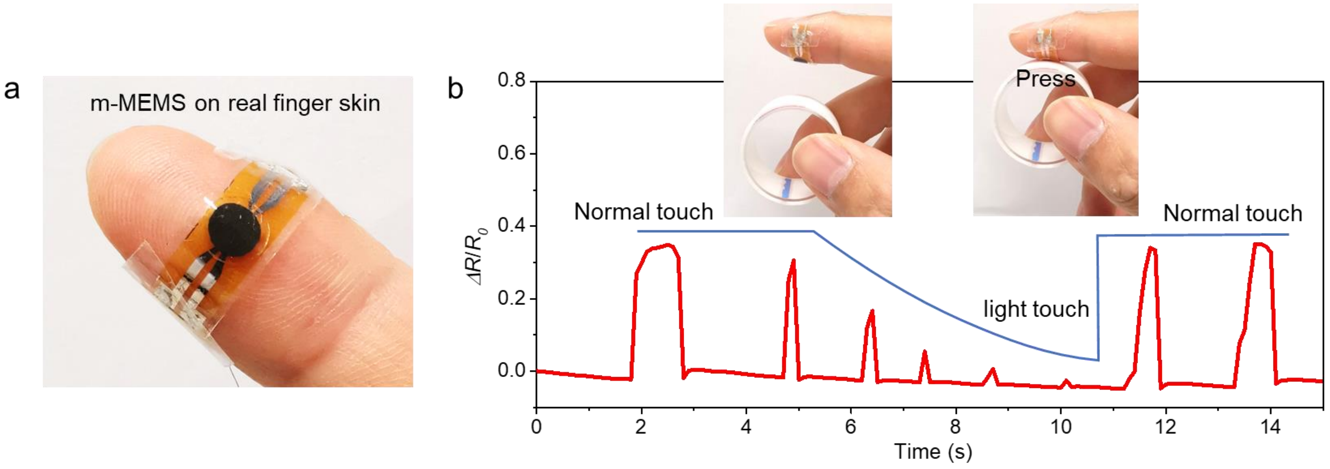

Supplementary Figure 22. m-MEMS worn on human finger. When applied to a soft substrate, like a fingertip, the shape deformation of the m-MEMS upon pressing will change the magnetic field at the position of the GMR sensor. Due to the soft nature of the human finger, this deformation will be different compared to the case when the sensor is applied to a rigid substrate like wooden finger. Still, conceptually/qualitatively nothing will change and the m-MEMS platform will act as a pressure sensor. This statement is supported by the experimental data shown in panel **b**. Here, we immobilized our m-MEMS platform on a finger (panel **a**). The resistance change of our sensor on the finger skin could distinguish the normal touch and light touch (panel **b** and Supplementary Movie 6). R_0 denotes the initial resistance of the m-MEMS platform. $\Delta R = R_0 - R$, where R is the real time resistance.

Supplementary Notes

Supplementary Note 1: Output voltage of the bridge

The output voltage of the bridge ($U_{\mathrm{out}}^{+} - U_{\mathrm{out}}^{-}$) is expressed as:

$$U_{\mathrm{out}}^{+} - U_{\mathrm{out}}^{-} = (U_{\mathrm{in}}^{+} - U_{\mathrm{in}}^{-})\left(\frac{1}{1+\frac{R_3}{R_4}} - \frac{1}{1+\frac{R_2}{R_1}}\right) \quad (1)$$

where, ($U_{in}^{+} - U_{in}^{-}$) is the input voltage, which is kept constant. Considering the arrangement, when the encircled sensor is located closer to the fingertip, it will be exposed to the strongest magnetic field of an external permanent magnet. In this case, when the MEMS platform with the Wheatstone bridge approaches the magnetic objects, the strength of the external magnetic field at the locations of the four GMR sensors has such relation: $H_1 > H_2 = H_3 > H_4$. Thus $R_2 = R_3 = R$ during the whole interaction processes. Therefore, the Supplementary Equation 1 can be rewritten as:

$$U_{\mathrm{out}}^{+} - U_{\mathrm{out}}^{-} = (U_{\mathrm{in}}^{+} - U_{\mathrm{in}}^{-})\left(\frac{1}{1+\frac{R}{R_4}} - \frac{1}{1+\frac{R}{R_1}}\right) \quad (2)$$

When the m-MEMS platform approaches a magnetic object, R_1 increases (due to the compensation of the external field via the bias magnetic field coming from the built-in magnetic field of the MEMS platform), while R and R_4 decrease (due to the peculiarity of the GMR effect where resistance decreases at higher magnetic field). In particular, considering a stronger magnetic field at the location of the sensors with the resistance R, this value decreases faster than R_4. Thus, upon approaching an external magnet, both the ratio R/R_4 and R/R_1 decrease. Still, as the ratio R/R_4 decreases slower than R/R_1, the difference in the bracket in the right-hand-side of the Supplementary Equation 2 will increase. Therefore, the output voltage ($U_{\mathrm{out}}^{+} - U_{\mathrm{out}}^{-}$) increases when the platform approaches magnetic objects.

When the platform gets in contact with the magnetic object and a pressing event takes place, R and R_4 will remain constant because of no displacement with respect to the source of the magnetic field. In contrast, R_1 will decrease because the built-in magnet moves closer to the built-in sensor. Thus, upon pressing, the ratio R/R_4 is constant while R/R_1 decreases. Therefore, the output voltage ($U_{\mathrm{out}}^{+} - U_{\mathrm{out}}^{-}$) decreases with the increase of pressure, which is confirmed by the experimental results in Fig. 5. The change of the signal of the sensing platform is illustrated in Supplementary Figure 18.

Supplementary Note 2: Angular sensing

The rotation of the sensor platform with respect to the external magnetic field leads to the compensation of the built-in magnetic field. In this respect, as illustrated in panel (c) of Supplementary Figure 21, the resistance of the compliant GMR sensor is determined by the absolute value of the total in-plane magnetic field. Taking the absolute magnetic field of the sensor and object being a and b, respectively, and θ - the rotation angle, the absolute value of the *total* magnetic field, $|H|_{\mathrm{total}}$, can be expressed as follows:

$$|H|_{\mathrm{total}} = \sqrt{a^2 + b^2 - 2ab\cos\theta}$$

With the increase (0 → 90°) or decrease (0 → -90°) of the rotation angle θ, the $|H|_{\mathrm{total}}$ will increase. Therefore, the resistance of the sensor decreases with the increase of the rotation angle (absolute

value). To demonstrate the rotation sensing functionality of the developed m-MEMS platform, we studied experimentally the rotation dependent resistance change of our sensor using a setup shown in panel (b) of Supplementary Figure 21. The signal evolution upon touchless interaction is shown in panel (c) of Supplementary Figure 21. The resistance of our sensor increases when the sensor approaches the object from about 7 cm to 3 cm. Then, we hold the sensor at the position of 3 cm and rotate the sensor from 0 to 50°. This corresponds to a typical range of angular displacement of a human pointing finger. We can see that the resistance of our sensor decreases ($\Delta R/R_0$ increases) versus the increase of the rotation angle. In panel (c) of Supplementary Figure 21, we can also see that the rotation-induced readout signal is still within the range assigned by the algorithm as being touchless interaction. Therefore, the signal of the rotation-based touchless interaction is also separated from the signal of the tactile interaction.

Supplementary References

1. S. Park *et al.*, Stretchable Energy-Harvesting Tactile Electronic Skin Capable of Differentiating Multiple Mechanical Stimuli Modes. *Adv. Mater.* 26, 7324-7332 (2014).
2. C. Pang *et al.*, A flexible and highly sensitive strain-gauge sensor using reversible interlocking of nanofibres. *Nat. Mater.* 11, 795-801 (2012).
3. X. Wang *et al.*, Silk-Molded Flexible, Ultrasensitive, and Highly Stable Electronic Skin for Monitoring Human Physiological Signals. *Adv. Mater.* 26, 1336-1342 (2014).
