# Supplementary material for: A bimodal soft electronic skin for tactile and touchless interaction in real time
Source: Nat Commun. 2019 Sep 27;10:4405. doi: 10.1038/s41467-019-12303-5 (PMC6764954; doi:10.1038/s41467-019-12303-5)
Supplement: Supplementary file 3 — Description of Additional Supplementary Files [file 41467_2019_12303_MOESM3_ESM.pdf]

Description of Additional Supplementary Files

File Name: Supplementary Movie 1
Description: Magnetic MEMS sensor interacts with a magnet-decorated petal. The evolution of the sensor signal upon touchless and tactile interaction is shown.

File Name: Supplementary Movie 2
Description: The movie demonstrates the presence of the turning point of the signal readout at the switching between tactile and touchless interaction

File Name: Supplementary Movie 3
Description: Animated mechanical simulation revealing the deformation of the m-MEMS platform under low and high pressure.

File Name: Supplementary Movie 4
Description: Compliant m-MEMS platform is applied to realize multichoice 3D touch for augmented reality applications.

File Name: Supplementary Movie 5
Description: Use of the compliant m-MEMS platform as angle sensor to determine direction is space.

File Name: Supplementary Movie 6
Description: Operation of the compliant m-MEMS platform in the tactile mode when applied to a pointing finger.
